# Letter to the editor: “Pelvic floor imaging with MR defecography: correlation with gynecologic pelvic organ prolapse quantification by Swamy et al.”

**DOI:** 10.1007/s00261-020-02609-0

**Published:** 2020-06-30

**Authors:** Julián Guerra, Maria Alejandra Rueda, Nelson Bedoya, Vanessa Murad

**Affiliations:** Fundación Santa Fe de Bogotá, Bogotá, Colombia

To the editor,

We read with pleasure the article: “Pelvic floor imaging with MR defecography: correlation with gynecologic pelvic organ prolapse quantification by Swamy et al.” [1].

The prolapse of the organs of the pelvic floor is a frequent pathology in women who have had children, both in the pre and postmenopause. Its diagnosis is not easy, but it is essential to determine the need for treatment and its type.

In our experience, physical examination and POP-Q are less and less used by gynecologists and proctologists because they are complex methods that require extensive experience, where the measurements performed can have great variability and poor correlation with reality, which can directly affect the surgical and clinical outcome of the patients.

For this reason, in our hospital and in our setting, magnetic resonance defecography (MRD) is increasingly recommended and used for the diagnosis and surgical planning of these patients. It is a complete diagnostic modality that allows an anatomical and functional analysis of the pelvic floor. In the anatomical evaluation, the different musculoskeletal components of the pelvic floor, the structures that limit each of the compartments and their contents can be identified in detail, to identify possible related pathologies. The dynamic component, where different measurements are made based on anatomical parameters that are easy to identify, which makes it highly reproducible and not very variable, allows evaluating the presence or absence of prolapse and determining its severity.

Multiple studies, including this one, have shown that there is a greater correlation between clinical and imaging findings for the anterior and middle compartments, compared to the posterior compartment. In our experience, there are three main imaging findings related to posterior compartment dysfunction, which are difficult to identify on physical examination or are even undetectable, and can hide or decrease the severity of the decrease in the anorectal junction and/or prolapse, explaining the poor correlation between the clinic and the images. These findings are: (a) the presence of hypertrophy of the puborectal muscle with indentation of the posterior wall of the rectum, (b) the presence of anterior rectocele, and (c) the paradoxical muscular contraction of the pelvic floor or anismus, represented by the measurement of the anorectal angle. Detecting these disorders and adequately describing them substantially improves the understanding of the pathology of the posterior compartment, guide the clinician and improve the surgical outcome of these patients [2].

On the other hand, we would like to mention our experience regarding the evaluation of pelvic floor dysfunction in men, for which we also use MRD frequently in the institution. The prevalence of pelvic disease in men is lower compared to female, finding as main risk factors in men age, muscular atrophy, obesity, prostatectomy, radiation, smoking and conditions that increase the intra-abdominal pressure (chronic constipation).

The anatomical evaluation in men differs in the interpretation of the compartments; here we evaluate an anterior compartment (genitourinary), a posterior compartment (anorectal), and the prostate, which is located under the bladder and surrounds the urethra. Other important anatomical structures in male pelvic floor include the urogenital diaphragm which is crossed by the membranous urethra and the deep dorsal vein of the penis through two separate openings, the pelvic diaphragm and the superficial perineal pocket. The dynamic component includes besides the measures of the anterior and posterior compartments, two important angles: the puboprostatic angle and the prostaticourethral angle. The puboprostatic angle is important because its increase is abnormal during Valsalva maneuver or defecation. The prostaticourethral angle is important because its increase in rest in patients with prostatic hyperplasia correlates with obstruction of urine output and urinary retention [3].

We highly appreciate the contributions of this study and we share that MRD is a highly sensitive and specific study for the evaluation of pelvic floor dysfunction in women. In addition, we highlight the importance of MRD in men to evaluate anatomical and dynamic components. There are few studies that focus on male pelvic anatomy and dysfunction, so we think that determining the standard measurements, their interpretation and correlation with clinical context, opens a huge window of opportunity.
